# Trainees between theoreticalknowledge and the initiative takingat the hospital

**DOI:** 10.1192/j.eurpsy.2022.2284

**Published:** 2022-09-01

**Authors:** I. Betbout, B. Amemou, A. Ben Haouala, W. Bouraoui, S. Chida, F. Zaafrane, A. Mhalla, L. Gaha

**Affiliations:** 1 fattouma bourguiba hospital, Psychiatry, Psychiatry Research Laboratory Lr05es10 “vulnerability To Psychoses” Faculty Of Medicine Of Monastir, University Of Monastir, monastir, Tunisia; 2 High school of sciences and techniques of the health of Sousse, Psychiatry, sousse, Tunisia

**Keywords:** practical knowledge, Patricia Benner, Initiative taking, coach

## Abstract

**Introduction:**

The internshipis a period in which the studentimplements what they have learned from their training to obtain or certification and to promote their professionalintegration, the difficulty of taking initiative and the lack of self-esteemrepresenting an obstacle to their training

**Objectives:**

This is a quantitative descriptive study conducted at the different placement departments among all 2nd-year students in all sections. Our data collection was done using two questionnaires administered, one for the supervisors and the other for the students.

**Methods:**

Theoretical Framework: Theorist Patricia Benner

**Results:**

According to the results found, in oursample, thereis a predominance of females 89.17%, with a sex ratio of 0.121. 88.34% are aged between 20 - 21 years and an averageage of 22.4 years. According to the interpretation of the Rosenberg Self-Esteem Scale scores, 17.5% of the trainees have a “Very Low Self-Esteem”, 47.5% have a “Low Self-Esteem”, 25.83% have an “Average Self-Esteem”, and only 9.17% have a “High Self-Esteem”. In addition, more than half of the respondents, 53.33%, state thatthey “often” have difficultytaking the initiative in the traineeshipenvironment, while 30.83% do not have such difficulty but “rarely”. Indeed, 53.33% of confirmedsupervisorssaythatthey “often” have difficultytaking the initiative in the placement environment

**Conclusions:**

It isnecessary to take into account these obstacles to the trainee’s training through better psychological supervision, which could be the first steptowardssolving the problem

**Disclosure:**

No significant relationships.

